# Evaluation of Medication-Related Osteonecrosis of the Jaw (MRONJ) Knowledge in Senior Dental and Medical Students

**DOI:** 10.7759/cureus.91640

**Published:** 2025-09-05

**Authors:** Tuba Develi, Merve Gaye Akgok, Madina Wardak

**Affiliations:** 1 Department of Oral and Maxillofacial Surgery, Istanbul Medipol University, Istanbul, TUR; 2 Department of Health Sciences, Istanbul Medipol University, Istanbul, TUR; 3 School of Dentistry, Istanbul Medipol University, Istanbul, TUR

**Keywords:** antiangiogenic therapy, antiresorptive therapy, bisphosphonate therapy, jaw osteonecrosis, medication-related osteonecrosis of the jaw (mronj)

## Abstract

Medication-related osteonecrosis of the jaw (MRONJ) is a complication that occurs in patients receiving antiresorptive or antiangiogenic medical therapy. Treatment of MRONJ requires meticulous treatment planning and protocols. The aim of this study is to evaluate and compare the knowledge level of final-year dental and medical students regarding MRONJ. A cross-sectional study was conducted on a total of 154 final-year students from the faculties of dentistry and medicine. Data were collected using a structured electronic survey consisting of six sections. Data were entered and analyzed using IBM SPSS Statistics for Windows, version 23, and a P-value <0.05 was considered significant. Only a few participants in the sample population had knowledge about antiresorptive or antiangiogenic drugs; the vast majority of those who were knowledgeable had been introduced to the topic during their university education. The underlying diseases that antiresorptive and antiangiogenic drugs target were unknown to the majority. Almost half of the sample could not identify any antiresorptive or anti-angiogenic drugs, and 45 (62.5%) senior students of the Faculty of Medicine and 13 (15.9%) senior students of Dentistry did not know that these drugs could cause jaw necrosis. Although the level of knowledge about MRONJ was higher among dental students across all departments in the survey, it was quite insufficient in both medical and dental students. Enhancing the knowledge of dentistry students and medicine students about MRONJ will be an important step in reducing and even preventing this public health problem, which is quite common in society.

## Introduction

Bisphosphonate-related osteonecrosis (BRONJ) was first described by Marx et al. in 2003 [1]. In 2009, the American Association of Oral and Maxillofacial Surgeons (AAOMS) defined BRONJ as exposed bone in the oral cavity for more than eight weeks in patients using bisphosphonates (BP) without a history of radiation therapy. Later, in 2014, the AAOMS changed the term to medication-related osteonecrosis of the jaw (MRONJ) to include the significant number of cases of osteonecrosis associated with other antiresorptive and antiangiogenic therapies [2,3-4].

MRONJ is associated with several medications, including but not limited to BP, denosumab, and antiangiogenic agents [5]. BPs bind to hydroxyapatites, inhibit bone remodeling, and have a very long half-life [6]. Denosumab is an inhibitor of nuclear factor kappa B ligand (RANKL). It inhibits osteoblast differentiation and function. Its half-life is shorter compared to BP. BPs and denosumabs are used to prevent bone metastasis in osteoporosis and cancer treatment [7]. Antiangiogenics are generally used to prevent new vessel formation in cases of malignancy. Examples include sunitib, sorafenib, and bevacizumab [8].

MRONJ manifests itself as exposed and lifeless bone in the maxillofacial structures following the use of intravenous or oral antiresorptive drugs [9,10-11]. Although more than 20 years have passed since MRONJ was first reported, the pathophysiology and pathology of MRONJ remain controversial. Definitive treatment strategies for MRONJ have not been developed. There are three main hypotheses explaining the pathophysiology of MRONJ [3,6]. The first hypothesis and the most accepted one is that it suppresses the turnover of bone by inhibiting osteoclastic activity and apoptosis [6]. The second hypothesis is that it disrupts the integrity of the oral mucosa through a direct toxic effect on epithelial cells and macrophages, leading to infection and subsequent bone necrosis. According to the third hypothesis, excessive bone resorption due to bacterial infection leads to osteonecrosis of the jaw [12].

The main risk factors for the development of MRONJ include those directly related to the drug, treatment indications, duration, dosage, and route of administration [13]. Other risk factors include dentoalveolar operations, anatomical factors, periapical pathologies, periodontal diseases, smoking, patients with comorbidities such as anemia and diabetes mellitus, and those receiving concurrent corticosteroid and chemotherapy treatment. The use of oral BP for benign bone diseases unrelated to malignancy, such as osteoporosis, osteopenia, Paget’s disease, and osteogenesis imperfecta, is associated with a lower risk of MRONJ, whereas long-term oral BP therapy, low-dose denosumab, intravenous BP, high-dose denosumab, or antiangiogenic agents, particularly when administered as part of oncologic treatment (e.g., metastatic bone disease), are associated with a higher risk of MRONJ [2]. Patients in this risk category are usually prescribed medications associated with MRONJ, either orally or intravenously [14,15]. The risk of developing MRONJ is low in patients who take oral antiresorptives for less than four years in cases unrelated to cancer and who do not have other clinical risk factors [2]. However, patients undergoing cancer treatment, especially conditions such as multiple myeloma and bone metastases, are at high risk [2,14-16].

Medication-related osteonecrosis of the jaw (MRONJ) remains a significant clinical challenge, and prevention plays a key role in reducing its incidence. Evidence suggests that providing predental treatment and oral hygiene motivation before initiating therapy yields better outcomes in patients at risk of developing MRONJ [2,3]. In this context, a multidisciplinary approach involving both medical doctors and dentists is considered essential. Despite its importance, this topic has not been sufficiently incorporated into dental and medical curricula, and there are only a few studies evaluating the knowledge levels of dental students and dentists regarding MRONJ [17-19]. These gaps underline the need for further investigation. 

Comparison and evaluation of MRONJ knowledge levels of dentistry and medicine students have not been conducted before in the literature. Identifying, addressing, and comparing knowledge gaps may contribute to the prevention of potential complications in patients at risk of MRONJ and may enable the development of educational programs. The aim of this study is to evaluate the knowledge levels of dentistry and medicine students about medication-related osteonecrosis of the jaw bones. The hypothesis of this study is that there is no difference in the level of knowledge between senior dentistry students and senior medicine students.

This article was previously posted to the Research Square preprint server on October 21, 2024 (https://doi.org/10.21203/rs.3.rs-5073401/v1).

## Materials and methods

This survey was conducted to evaluate and compare the knowledge of final-year dental students and final-year medical students at Istanbul Medipol University regarding MRONJ. This study was approved by the institutional ethics committee of Istanbul Medipol University and was conducted in accordance with the ethical standards defined in the Declaration of Helsinki (no. 10840098-772.02-7719). The survey was conducted between January and May 2023 with 157 participants, including senior dentistry and medicine students from Istanbul Medipol University. Three participants were excluded from the study due to incomplete responses. Group one (senior dentistry students 82), Group two (senior medicine students 72). All participation in the study was voluntary, and written informed consent was obtained from the participants before completing the questionnaire. Participants were selected using a nonprobability purposive sampling technique, and the data were collected using a validated self-administered structured questionnaire. Approval to use the questionnaire was obtained from a published article [17]. The survey was later modified [3]. The modified survey has been updated for our own research. The survey instrument utilized in this study can be accessed in the Appendix.

The survey is organized into six parts. The first part collects general demographic information, including age, gender, and university. The second part consists of six questions concerning prior knowledge about antiresorptive and antiangiogenic medications and the participant’s assessment of the significance of this information. The third section includes five questions that assess the participant’s understanding of the therapeutic applications of these medications. The fourth section contains three questions focused on the participant’s knowledge of osteonecrosis of the jaw and its associated risks. The fifth section features four questions about the dental management of patients receiving medications associated with MRONJ, including BPs, denosumab, antiresorptive agents, and antiangiogenic agents. The final section, comprising three questions, investigates how frequently participants encounter patients with osteonecrosis of the jaw, with or without such medications, in their clinical practice.

Data collection was carried out using a structured electronic survey. Data were entered and analyzed using IBM SPSS Statistics for Windows, Version 23.0 (released 2015, IBM Corp., Armonk, NY). The Shapiro-Wilk test was used to determine the distribution of the data. The Chi-square test was used to compare categorical variables and evaluate the demographic distribution between the two faculties and the effects of certain factors on their relationships. In statistical analyses, a significance level of p < 0.05 was adopted; accordingly, for the results to be considered significant, the p-value must be less than 0.05.

## Results

Our hypothesis was rejected. Of the 157 participants who participated in the study, three participants were excluded from the study because they did not complete the questions. Of the 154 participants, 82 (53%) were senior dentistry students, and 72 (47%) were senior medicine students. Fifty-four (65.9%) dentistry students were female, 28 (34.1%) were male, and 40 (55.6%) medicine students were female, while 32 (44.4%) were male.

Table 1 shows the details of the survey responses regarding receiving prior information and perception of the importance of information. In both groups, universities were identified as the main source of information regarding antiresorptive and antiangiogenic drugs. Seventy-nine (96.3%) Faculty of Dentistry students and 52 (72.2%) Faculty of Medicine students stated that it is important to ask questions about the patient’s use of antiresorptive/antiangiogenic drugs during the examination. There is a statistically significant difference between the two groups.

**Table 1:** General knowledge of antiresorptive/antiangiogenic medications * p < 0.05

Questions	Faculty				P
	Medicine		Dentistry		0.209
Where did you hear about antiresorptive drugs?	n	%	N	%	
I have never heard of it.	17	23.60%	9	11.00%	
University	51	70.80%	69	84.10%	
Social media	1	1.40%	0	0.00%	
Scientific journals	1	1.40%	2	2.40%	
Other	2	2.80%	2	2.40%	
Where did you hear about antiangiogenic drugs?					0.098
I have never heard of it.	16	22.20%	8	9.80%	
University	48	66.70%	69	84.10%	
Social media	1	1.40%	0	0.00%	
Scientific journals	4	5.60%	4	4.90%	
Other	3	4.20%	1	1.20%	
Do you think it is important to ask whether patients are using antiresorptive/antiangiogenic medications?					0.000*
Yes	52	72.20%	79	96.30%	
No	20	27.80%	3	3.70%	

Table 2 shows questions and answers about antiresorptive and antiangiogenic drugs. When the relationship between the two groups and the diseases in which antiresorptive drugs are used, there is a statistically significant difference between the groups with bone metastasis, osteomyelitis, multiple myeloma, Paget's, and not knowing the answer. When looking at the indications for the use of antiresorptive drugs for bone metastasis, osteomyelitis, multiple myeloma, and Paget's disease, there is a significant difference between the knowledge level of the two groups. The level of knowledge about these disorders is high among dentistry students.

**Table 2:** Knowledge of therapeutic uses of antiresorptive/antiangiogenic medication * p < 0.05

Questions	Faculty				P-value
	Medicine		Dentistry		
What diseases are targeted by antiresorptive therapy?	n	%	N	%	
Bone metastases	16	22.20%	42	51.20%	0.000*
Osteomyelitis	12	16.70%	42	51.20%	0.000*
Multiple myeloma	23	31.90%	40	48.80%	0.025*
Hypercalcemia of malignancy	21	29.20%	27	32.90%	0.372
Osteopetrosiss	21	29.20%	30	36.60%	0.211
Osteopenia	28	38.90%	22	26.80%	0.078
Chondroblastoma	6	8.30%	6	7.30%	0.524
Osteogenesis imperfecta	13	18.10%	20	24.40%	0.224
Paget's disease of bone	14	19.40%	35	42.70%	0.002*
I do not know	28	38.90%	13	15.90%	0.001*
What diseases are targeted by anti-angiogenic therapy?					
Elastofibromas	3	4.20%	13	15.90%	0.015*
Metastatic colorectal cancer	17	23.60%	19	23.20%	0.549
Leiomyomas	5	6.90%	13	15.90%	0.070
Renal cell cancer	21	29.20%	15	18.30%	0.081
Neuroendocrine tumor of the pancreas	22	30.60%	11	13.40%	0.008*
Multiple myeloma	16	22.20%	21	25.60%	0.382
Granular cell tumors	14	19.40%	14	17.10%	0.431
I do not know	39	54.20%	47	57.30%	0.409
Mark the name of the antiresorptive drugs you are familiar with:					
I do not know of any antiresorptive drug	25	34.70%	31	37.80%	0.410
Alendronate	18	25.00%	16	19.50%	0.266
Risedronate	9	12.50%	15	18.30%	0.222
Ibandronate	12	16.70%	21	25.60%	0.124
Neridronate	11	15.30%	10	12.20%	0.373
Pamidronate	13	18.10%	25	30.50%	0.054
Zolendronate	20	27.80%	45	54.90%	0.001*
Tiludronate	7	9.70%	20	24.40%	0.014*
Denosumab	12	16.70%	29	35.40%	0.007*
Mark the name of the anti-angiogenic drugs you are familiar with:					
I do not know of any anti-angiogenic drugs	37	51.40%	55	67.10%	0.035
Sunitib	16	22.20%	13	15.90%	0.211
Sorafenib	28	38.90%	25	30.50%	0.177
Bevacizumab	24	33.30%	10	12.20%	0.001*
Sirolimus	9	12.50%	7	8.50%	0.294
Do you know that anti-resorptive/antiangiogenic medications can lead to osteonecrosis of the jaw?					
Yes	27	37.50%	69	84.10%	0.000*
No	45	62.50%	13	15.90%	

Considering the indications of antiangiogenic drugs, there is a significant difference between the groups for elastofibroma and pancreatic neuroendocrine tumors. When it comes to elastofibroma, dentistry students are more dominant, while medical students are more dominant for pancreatic neuroendocrine tumors. When the relationship between known antiresorptive drugs and the groups is examined, a significant difference was found between the groups in terms of knowledge of the drugs Zolendronate, Tiludronate, and Denosumab. It is seen that the knowledge level of dentistry students is higher for these three drugs, but it is still insufficient. When the relationship between known antiangiogenic drugs and the groups is examined, the known status of bevacizumab and groups, there is a significant relationship between. It appears that the knowledge level of medical students is better regarding this drug. There is a significant difference between the groups in terms of knowing that antiresorptive/antiangiogenic drugs can cause osteonecrosis of the jaw. Although the knowledge level of dentistry students is higher, there is still a need to increase their knowledge level for public health.

Table 3 shows the risk factors related to osteonecrosis of the jaw. When the relationship between the stated risk factors for jaw osteonecrosis and the faculties is examined, smoking, alcohol, treatment duration, steroid treatment, amount of medication, microtrauma, and not knowing the answer have a significant relationship with the groups.

**Table 3:** Knowledge of risk factors related to osteonecrosis of the jaw * p < 0.05

Questions	Faculty				P-value
	Medicine		Dentistry		
Which of the following are the risk factors related to osteonecrosis of the jaw?	n	%	N	%	
Tobacco	43	59.70%	62	75.60%	0.026*
Antibiotic therapy	12	16.70%	16	19.50%	0.404
Alcohol	27	37.50%	48	58.50%	0.007*
Arterial hypertension	12	16.70%	17	20.70%	0.332
Length of therapy	28	38.90%	55	67.10%	0.000*
Hyperlipidemia	12	16.70%	19	23.20%	0.211
Steroid therapy	29	40.30%	47	57.30%	0.025*
Total amount of drug administered	25	34.70%	63	76.80%	0.000*
Micro-trauma	20	27.80%	41	50.00%	0.004*
I do not know.	22	30.60%	4	4.90%	0.000*

Table 4 presents the questions and answers regarding dental treatment in patients receiving medications associated with the development of MRONJ. There is a statistically significant difference between patients’ opinions that they should be checked by dentists before starting BP treatment. It seems that the medicine faculty students have less awareness. There is a significant difference between the groups in the answers given to questions about whether invasive dental treatments can be safely applied to patients who are or will be treated with intravenous and oral BP medication. Although dental students demonstrated a higher level of knowledge compared to medical students, both groups require further improvement.

**Table 4:** Knowledge of dental management in patients undergoing bisphospate therapy * p < 0.05

Questions	Faculty				P-value
	Medicine		Dentistry		
Do you think patients should be checked by the dentist before starting an IV bisphosphonate treatment?	n	%	N	%	
Yes	54	78.30%	80	97.60%	0.000*
No	15	21.70%	2	2.40%	
I do not know.	0	0.00%	0	0.00%	
Can invasive dental treatments be performed safely on patients during intravenous bisphosphonate drug therapy?					0.000*
Yes	3	4.20%	13	15.90%	
No	16	22.20%	62	75.60%	
I do not know.	53	73.60%	7	8.50%	
Can invasive dental treatments be performed safely on patients using oral bisphosphonates for <4 years without risk factors?					0.000*
Yes	14	19.40%	70	85.40%	
No	5	6.90%	6	7.30%	
I do not know.	53	73.60%	6	7.30%	
Can invasive dental treatments be performed safely on patients using oral bisphosphonates for <4 years with risk factors?					0.000*
Yes	5	6.90%	20	24.40%	
No	11	15.30%	51	62.20%	
I do not know.	56	77.80%	11	13.40%	
Can invasive dental treatments be performed safely on patients using oral bisphosphonates for >4 years?					0.000*
Yes	4	5.60%	10	12.20%	
No	14	19.40%	65	79.30%	
I do not know.	54	75%	7	8.50%	

Table 5 includes the survey results examining the relationship between the presence of jaw osteonecrosis and antiresorptive drug use and treatment.

**Table 5:** Knowledge of jaw osteonecrosis and antiresorptive drug use and treatment * p < 0.05

Questions	Faculty				P-value
	Medicine		Dentistry		0.047*
Have you treated patients with osteonecrosis of the jaw and are on antiresorptive therapy?	n	%	N	%	
Yes	1	1.40%	7	8.50%	
No	71	98.60%	75	91.50%	
Have you treated patients with osteonecrosis of the jaw and are NOT on antiresorptive therapy					
Yes	52	2.80%	14	17.10%	0.003*
No	70	97.20%	68	82.90%	
Have you treated patients without osteonecrosis of the jaw and are on anti-resorptive therapy?					
Yes	2	2.80%	12	14.60%	0.009*
No	70	97.20%	70	85.40%	

## Discussion

MRONJ is a serious adverse effect linked to long-term antiresorptive or antiangiogenic drug therapies, primarily impacting the bones of the jaw. Having sufficient knowledge about MRONJ is of great importance to improve treatment results, prevent this problem and reduce complications [2]. It has been observed that 99.7% of dentists do not have sufficient knowledge about diagnosing and managing MRONJ [20]. Another study showed that 60% of the participants had a good level of knowledge about MRONJ. While 50% of the participants hesitated to treat patients using BP, 63% stated that they could refer patients using BP [21]. In a study comparing the knowledge levels of dentists and dental students, it was found that 62% of dentists and 58% of dental students had insufficient knowledge of MRONJ [18]. According to the study measuring the knowledge level of dentists and dental students, 50% of the students and 68% of the dentists have up-to-date information about MRONJ. Most correctly identified the relevant risk factors. 13% of the students and 33% of the dentists knew how to treat osteonecrosis when it occurred [19]. Another study in the literature showed that 99% of the participants knew BPs and that sixth-semester students had better knowledge than fourth-semester students [17]. On the contrary, another study showed that knowledge about the side effects of antiresorptive drugs decreased as the years of professional practice increased [22]. Another study showed that oral maxillofacial surgeons had more knowledge than general dentists [23]. According to a study conducted in Saudi Arabia in 2020, 60% of the participants were found to have poor BP knowledge [24]. According to a study conducted on medical students, almost all of the students stated that comprehensive dental screening was necessary and essential before starting BP use. 16% of the students answered correctly about the drugs that cause MRONJ [25]. In our study, senior dentistry students had 84.1% knowledge about MRONJ, while medical students had 37.5% knowledge. There is a significant statistical difference between faculties.

However, although studies investigating the level of knowledge about MRONJ among dental students are limited, there is no study comparing the level of knowledge of dental students and medical students about MRONJ. Generally, existing studies in the literature have focused on BPs. At this point, it is important to conduct a comprehensive study to evaluate the knowledge level of dental and medical students about MRONJ, improve their education, and enable them to manage these potential complications more effectively. Such a study could fill knowledge gaps in this field by identifying deficiencies in the diagnosis and treatment of MRONJ and contribute to the creation of a more effective training program for future dentists and medical practitioners. According to the research results, the level of knowledge about antiresorptive drugs shows a significant difference between the two faculties. However, interestingly, a lack of knowledge about antiresorptive drugs was detected in both faculties. This highlights the need for more comprehensive teaching and learning in both faculties, given the importance of this group of drugs.

Having deeper knowledge of issues such as the effects, side effects, and indications for the use of antiresorptive drugs can increase the professional competencies of medical and dental students. In this context, updating training programs and adopting an interdisciplinary approach that emphasizes the role of antiresorptive drugs in clinical practice may better equip future healthcare professionals on this important issue. These efforts will be an important step in terms of drug safety and best management of patients. The knowledge level of medical students that these drugs can cause osteonecrosis was determined as 33%. This low level of knowledge shows that the potential risks and side effects of this drug group are not well known by students.

One of the most important results is that the rate of thinking that a dentist should be consulted before starting BP treatment is 20% among medicine students. This approach is considered a preventive treatment to prevent the occurrence of MRONJ in advance, saving time and resources in this process for both the patient and the treatment cost. Therefore, the adoption of this preventive approach by medical school students should be considered a critical step in minimizing the risks associated with MRONJ.

It has been observed that dentistry students generally have good knowledge of drug relations in areas such as bone metastasis, osteomyelitis, multiple myeloma, and Paget's, but they have deficiencies in subjects they encounter less frequently, such as neuroendocrine tumors. On the other hand, it has been observed that medical students have better knowledge about some antiangiogenic drugs. Since it is not expected to provide specific and in-depth knowledge in this field other than the questions discussed here, it is not considered as important as the previous questions.

As a result, it is necessary to increase the awareness of MRONJ among faculty of medicine students and take preventive measures such as directing patients to the dentist, especially before starting this medication, performing oral and dental examination of the patients by the dentist, and protecting and intervening in their health. In particular, it is important to remember that MRONJ is the gold standard in prevention to avoid this troublesome and protracted complication. The need to strengthen information and education programs for students by increasing awareness in this field and emphasizing these preventive strategies comes to the fore. Addressing this deficiency may help future healthcare professionals guide their patients more effectively and minimize such complications.

Although the general level of knowledge about MRONJ was higher among dental students for all departments in the survey, it was quite inadequate for both medical and dental students. This lack of information may result in necessary treatments not being implemented, delayed, or affected. Nonetheless, a more significant concern is that clinicians may fail to recognize the risks when managing patients with MRONJ. These findings ultimately highlight the need for continuing education to improve patient care. These issues should be given more attention in the education of physician candidates.

Limitations

In addition, we cannot be certain about how carefully and honestly the participants responded during the survey. Since the study only included senior dentistry and medical students from Istanbul Medipol University, caution should be exercised when generalizing the findings. The survey should be repeated in a broader population and compared with students from different universities.

## Conclusions

Increasing MRONJ awareness, taking effective preventive measures, and being able to provide treatment when necessary are crucial for both individual and public health. In this regard, incorporating the missing aspects of MRONJ into the curricula of medical and dental faculties will not only improve students' understanding of the topic but also make future healthcare professionals more aware of it. These educational steps play a critical role in protecting public health and minimizing the societal impact of this disease. Furthermore, ensuring that future healthcare professionals have a more comprehensive understanding of MRONJ will help speed up treatment processes and prevent complications. As a result, the prevalence of MRONJ in the community is expected to decrease significantly.

## Appendices

**Table 6:** Modified survey

1	Age?
	A) 19-20 B) 21-22 C) 23-24 D) 25 or above
2	Gender?
	A) Male B) Female
3	In what year did you enroll in university?
	A) 2016 B) 2017 C) 2018 D) 2019
4	Select the faculty you studied at.
	A )Faculty of Dentistry B) Faculty of Medicine
5	Have you encountered any antiresorptive drugs such as bisphosphonates during your education?
	A) Yes B) No
6	Have you encountered any antiangiogenic drugs such as bisphosphonates during your education?
	A) Yes B) No
7	Where have you heard about antiresorptive drugs?
	A) Never heard of it B) University C) Mass media D) Scientific journals E) Other
8	Where did you hear about antiangiogenic drugs?
	A) Never heard of it B) University C) Mass media D) Scientific journals E) Other
9	Do you think it is important to ask whether patients are using antiresorptive/antiangiogenic medications?
	A) Yes B) No
10	In which diseases are antiresorptive drugs used? (more than one option can be selected)
	A) Bone metastasis B) Osteomyelitis C) Multiple Myeloma D) Malignant Hypercalcemia E) Osteopetrosis F) Osteopenia G) Chondroblastoma H) Osteogenesis Imperfecta I) Paget
11	In which diseases are antiangiogenic drugs used? (more than one option can be selected)
	A) Elastofibroma B) Metastatic Colorectal Cancer C) Leiomyoma D) Renal Cell Carcinoma E) Pancreatic Neuroendocrine Tumors F) Multiple Myeloma G) Granular Cell Tumor
12	Please select the antiresorptive drugs you know.
	A) I do not know any antiresorptive drugs B) Alendronate (Fosamax) C) Risedronate (Antonel) D) Ibandronate (Boniva) E) Neridronate (Nerixia) F) Pamidronate (Aredia) G) Zolendronate (Zometa) H) Tiludronate (Skelid) I) Denosumab (Prolia)
13	Please select the antiangiogenic drugs you know. (more than one option can be selected
	A) I do not know any antiangiogenic drugs. B) Sunitinib (Sutent) C) Sorafenib (Nexavar) D) Bevacizumab (Avastin) E) Sirolimus (Rapamune)
14	Did you know that antiresorptive/antiangiogenic medications can cause osteonecrosis of the jaw?
	A) Yes B) No
15	Which of the following are risk factors for osteonecrosis of the jaw? (more than one option can be selected)
	A) Cigarette B) Antibiotic treatment C) Alcohol D) Hypertension E)Treatment duration F) Hyperlipidemia G) Steroid treatment H) Amount of medicine i) Micro trauma
16	Do you think patients should be checked by dentists before starting IV bisphosphonate therapy?
	A) Yest B) No
17	Can invasive dental treatments be safely performed on patients during intravenous bisphosphonate drug therapy?
	A) Yes B) No C) I do not know
18	Can invasive dental treatments be safely applied to patients who have used oral bisphosphonates for less than 4 years and have no risk factors?
	A) Yes B) No C) I do not know
19	Oral bifosfonatları 4 yıldan az kullanan ve risk faktörlerine sahip hastalarda invaziv diş tedavileri güvenle uygulanabilir mi?
	A) Yes B) No C) I do not know
20	Can invasive dental treatments be performed safely in patients who have used oral bisphosphonates for less than 4 years and have risk factors?
	A) Yes B) No C) I do not know
21	Have you ever treated patients with osteonecrosis of the jaw who were receiving antiresorptive therapy?
	A) Yes B) No
22	Have you ever treated patients with osteonecrosis of the jaw who were not receiving antiresorptive therapy?
	A) Yes B) No
23	Have you ever treated patients without osteonecrosis of the jaw who were receiving antiresorptive therapy?
	A) Yes B) No
